# HLA-E-Restricted Cross-Recognition of Allogeneic Endothelial Cells by CMV-Associated CD8 T Cells: A Potential Risk Factor following Transplantation

**DOI:** 10.1371/journal.pone.0050951

**Published:** 2012-11-30

**Authors:** Mathilde Allard, Pierre Tonnerre, Steven Nedellec, Romain Oger, Alexis Morice, Yannick Guilloux, Elisabeth Houssaint, Béatrice Charreau, Nadine Gervois

**Affiliations:** 1 Inserm, U892, Nantes, France; 2 Univ Nantes, Nantes, France; 3 CNRS, UMR 6299, Nantes, France; 4 Inserm, U1064, Nantes, France; 5 CHU Nantes, Nantes, France; Beth Israel Deaconess Medical Center, Harvard Medical School, United States of America

## Abstract

Although association between CMV infection and allograft rejection is well admitted, the precise mechanisms involved remain uncertain. Here, we report the characterization of an alloreactive HLA-E-restricted CD8 T cell population that was detected in the PBL of a kidney transplant patient after its CMV conversion. This monoclonal CD8 T cell population represents a sizable fraction in the blood (3% of PBL) and is characterized by an effector-memory phenotype and the expression of multiple NK receptors. Interestingly, these unconventional T cells display HLA-E-dependent reactivity against peptides derived from the leader sequences of both various HCMV-UL40 and allogeneic classical HLA-I molecules. Consequently, while HLA-E-restricted CD8 T cells have potential to contribute to the control of CMV infection *in vivo*, they may also directly mediate graft rejection through recognition of peptides derived from allogeneic HLA-I molecules on graft cells. Therefore, as HLA-E expression in nonlymphoid organs is mainly restricted to endothelial cells, we investigated the reactivity of this HLA-E-restricted T cell population towards allogeneic endothelial cells. We clearly demonstrated that CMV-associated HLA-E-restricted T cells efficiently recognized and killed allogeneic endothelial cells *in vitro*. Moreover, our data indicate that this alloreactivity is tightly regulated by NK receptors, especially by inhibitory KIR2DL2 that strongly prevents TCR-induced activation through recognition of HLA-C molecules. Hence, a better evaluation of the role of CMV-associated HLA-E-restricted T cells in transplantation and of the impact of HLA-genotype, especially HLA-C, on their alloreactivity may determine whether they indeed represent a risk factor following organ transplantation.

## Introduction

Cytomegalovirus (CMV) is a common opportunistic pathogen that persists for life in the human host after primary infection. While CMV infection of immunocompetent individuals generally results in subclinical diseases, it may cause serious life threatening complications in immunocompromised ones. Consequently, transplant patients with immunosuppressive regimens are particularly prone to CMV disease and it is estimated that up to 75% of all patients undergoing solid organ transplantation experience new infection or reactivation of latent CMV infection [1], [2].

CMV infection has been implicated in the development of both acute and chronic allograft rejection and has been associated with decreased allograft and patient survival [3], [4]. Although association between CMV infection and allograft rejection is well admitted, the precise mechanisms involved remain uncertain.

CMV could account for graft rejection by triggering the activation of endothelial cells, which are preferential targets of CMV infection [5], [6], [7]. This might directly increase the expression of MHC, costimulatory and adhesion molecules on the allograft endothelium through the induction of mediators release such as type I IFN. Then, activated graft’s EC may attract and activate recipient’s cytotoxic T cells, which can trigger rejection [8]. CMV infection could also be implicated in the development of allograft rejection because of cross-reactivity of CMV-specific T cells toward allogeneic HLA molecules as we and others have previously documented [9], [10], [11], [12]. An alternate mechanism has been suggested by studies reporting the existence in CMV seropositive individuals of CD8 T cells that recognize, in a HLA-E restricted-fashion, peptides derived from the leader sequences of both HCMV-UL40 and allogeneic classical HLA-I molecules [13], [14], [15], [16]. Consequently, while this HLA-E-restricted T cells potentially mediate protection against CMV infection, they may also promote graft rejection through recognition of peptides derived from allogeneic HLA-I molecules presented by HLA-E on graft cells.

One of the most striking features of the non-classical HLA-I molecule HLA-E is its highly conserved nature. Only two allelic forms exist in the Caucasian population, HLA-E*0101 (HLA-E^107R^) and HLA-E*0103 (HLA-E^107G^) that differ at one amino acid position [17]. As a consequence, HLA-E-bound peptides are highly restricted, comprising mostly signal peptides derived from others HLA-I proteins [18]. Class Ib molecules are often considered to have a prominent role in innate immunity. Among this line, surface expression of HLA-E bound to autologous HLA class I derived peptides, indicating the integrity of the MHC I antigen processing machinery and acting as a ligand for CD94-NKG2 receptors, modulate the activation of NK and T cells [19], [20]. However, in times of cellular stress or infections, HLA-E is associated with a much more diverse repertoire of peptides, which can be sensed directly by αβ TCR [21], [22]. Indeed, several studies in human and mice have highlighted a dual role for unclassical HLA-Ib molecules, in that, like classical HLA-Ia molecules (ie HLA-A/-B/-C), they can mediate adaptative immune responses to bacteria [23], [24], viruses [13], [25], [26], tumors [27] and self-antigens [28], [29].

Although HLA-E is virtually expressed in all tissues, its surface expression profile is more restricted than that of classical HLA-I molecules. We previously reported that, HLA-E surface expression in normal nonlymphoid organs is mainly restricted to endothelial cells [30]. Upon solid organ transplantation, because graft endothelial cells display MHC-peptide complexes at their surface and come in regular contact with recipient circulating T cells, the endothelium of allografts plays a central role in eliciting immune-mediated rejection [8], [31]. However, while HLA-E has been shown to behave as a strong transplantation antigen in rodent models [32], whether HLA-E expressed on human graft’s tissues could trigger an allogeneic cellular response remains to be documented.

Hence, the purpose of our study was to investigate the potential alloreactivity of CMV-associated HLA-E-restricted CD8 T cells isolated from a CMV seropositive recipient. We clearly demonstrated the reactivity of CMV-associated HLA-E-restricted CD8 T cells against allogeneic endothelial cells from transplant donors and its tight regulation by NK receptors and therefore address their potential involvement in human adaptative response against allograft organs.

## Materials and Methods

### HLA-E-restricted CD8 T Cells Isolation and Culture

Blood sample was collected from a CMV-seropositive kidney-transplant patient (HLA-A*0201, -B*4402, -B*5101, -Cw*0501 and -Cw*1402) (referred as KR2 in a previous study) [33] with formal consent. PBMC were isolated by a Ficoll density gradient (PAA, Les Mureaux, France) and cutured with RPMI 1640 (Sigma-Aldrich, Saint-Quentin Fallavier, France) containing 8% human serum (local production) and 150 U/mL rIL-2 (Eurocetus, Rueil-Malmaison, France). HLA-E-reactive population was enriched using a TNF-α Secretion Assay Cell Enrichment and Detection Kit (Miltenyi, Paris, France) after stimulation with HLA-E-transfected COS-7 cells. Sorted cells were cloned by limiting dilution and expanded by stimulation with phytohemagglutinin (PHA)-L (Sigma-Aldrich) in the presence of irradiated feeder cells (allogeneic lymphocytes and Epstein Barr Virus-transformed B lymphocytes) [34].

### HAEC Isolation, Culture and IFN-γ Activation

Human arterial endothelial cells (HAEC) were isolated from unused artery pieces collected at the time of kidney transplantation, harvested according to good medical practice and stored in the DIVAT Biocollection (French Health Minister Project number 02G55) [35]. All patients who participated in this study signed an informed consent and the study was performed according to the guidelines of the local ethics committee (CCPRB, CHU Nantes, France). Briefly, fragment of arteries were incubated with collagenase A (Roche, Basel, Switzerland) for 30 min at 37°C and EC were selected using CD31-Dynabeads (Dynal, Villebon sur Yvette, France). HAEC were grown in Endothelial Cell Basal Medium (ECBM) supplemented with 10% fetal calf serum (FCS, PAA, France), 0.004 m-L/mL ECGS/Heparin, 0.1 ng/mL hEGF, 1 ng/mL hbFGF, 1 µg/mL hydrocortisone, 50 µg/mL gentamicin and 50 ng/mL amphotericin B (C-22010, PromoCell, Heidelberg, Germany). For activation, confluent HAEC monolayers were starved overnight in ECBM supplemented with 2%FCS without growth factors and incubated with recombinant human IFN-γ (50 U/mL, Imukin, Boehringer Ingelheim, Germany) for 48 h. HLA class I genotyping was performed by the Etablissement Français du Sang (Nantes, France).

### B-EBV 721.221 and COS-7 Cells Culture

The HLA-E-transfected (721.221-E) and untransfected (721.221) B-EBV cell lines were kindly provided by V. Braud (UMR CNRS 6097/Université Nice-Sophia Antipolis, Valbonne, France) [36]. COS-7 cells were obtained from T. Boon (Ludwig institute for Cancer Research, Brussels, Belgium) [37]. These cells were maintained in RPMI 1640 10%FSC.

### Antibodies

The following antibodies were used in a conjugated form (phenotyping) or not (blocking or redirected lysis experiments) with fluorescein isothiocyanate (FITC), phycoerythrin (PE) or allophycocyanin (APC): TCRαβ-PE, CD8α-PE, IFN-γ-PE (Miltenyi), CD3-PE, CD27-PE, CD28-PE, CD45-RA-PE, CD45-RO-PE, CD56-PE, CD57-FITC, CD62-L-PE, CCR7-PE, CD107a-PE, Perforine-FITC, Granzyme-A-FITC, TNF-α-PE, GM-CSF-PE, TGF-β-PE, IL-2-PE, IL-4-PE, IL-5-PE, IL-13-PE, IL-21-PE, HLA-A/B/C (clone G46-2.6) (Becton Dickinson, Le Pont de Claix, France), CD8β-PE, CD94-PE (clone HP-3B1), NKG2A-PE (clone Z199), KIR2DS1/2DL1-APC (clone EB6), KIR2DS2/2DL2/2DL3-APC (clone GL183), KIR2DS4 (clone FES172), KIR3DS1/3DL1-PE (clone ZIN273), ILT-2-PE (clone HPF1) (Beckman Coulters, Villepinte, France), NKG2C-PE (clone 134522), NKG2D-PE (clone 149810), IL-17F-PE (R&D, Lille, France), IL-22-PE, HLA-E (clone 3D12) (BioLegend, San Diego, CA) and HLA-I (clone W6.32, American Type Culture Collection).

### Peptides and Recombinant Peptide/HLA-E Monomers

Peptides VMAPRTLLL, VMAPRTLVL, VMAPRTVLL and VMAPRTLIL (HLA-A*01-, HLA-A*02-, HLA-B*07- and HLA-Cw*01-derived signal peptides respectively) with purity >85% were purchased from Eurogentec (Angers, France). HLA-E*0101/peptide monomers were generated by the recombinant protein facility of SFR26 (Nantes, France).

### Phenotypic Characterization by Flow Cytometry

For membrane staining, 2×10^5^ cells were incubated at 4°C with 10 µg/ml of Ab (specific or isotype control) or tetramers for 30 min or 1 h respectively. When non-conjugated mAb were used, a second incubation with PE-conjugated goat F(ab’)2 fragment anti-Mouse IgG (Beckman Coulters) was performed. 5×10^4^ cells were acquired in the viable cells gate on a FACScalibur flow cytometer using CellQuest software (Becton Dickinson). Relative fluorescence intensity (RFI) was calculated as sample mean fluorescence divided by isotype control mean fluorescence.

### Transient Transfection of COS-7 Cells and TNF Assay

Briefly, 20×10^3^ COS-7 cells were transfected with 100 ng of HLA-E*0101 or HLA-E*0103 encoding plasmid by the DEAE-dextran-chloroquine method. 48 h after transfection, 5×10^3^ T cells were added to transfected COS-7 cells. Culture supernatants were harvested 6 h later and tested for TNF content through assessment of the sensitive WEHI164 clone13 viability in a MTT colorimetric assay.

### Intracellular Staining

For cytokine/perforine/granzyme intracellular staining, 1×10^5^ T cells were stimulated in the presence of Brefeldin A (Sigma-Aldrich, 10 µg/ml) with 2×10^5^ target cells (B-EBV cells or HAEC) for 6 h at 37°C, in the presence or not of blocking Abs. For peptide loading, target cells were incubated with peptides for 1 h at 37°C before incubation with T cells. Cells were then fixed with 4% paraformaldehyde (Sigma-Aldrich), labeled with specific mAbs and analyzed by flow cytometry.

### CD107a Degranulation

1×10^5^ T cells were stimulated with 2×10^5^ target cells in the presence of anti-CD107a mAb. After 4 h at 37°C, cells were analyzed by flow cytometry.

### TCR-αβ/CD3/CD8 Downregulation

1×10^5^ T cells were stimulated with 2×10^5^ target cells at 37°C. After the indicated time, TCR-αβ/CD3/CD8 fluorescence intensity was measured in unstimulated and activated lymphocytes. Data were expressed as percentages of RFI that were calculated according to the following formula: (RFI of activated lymphocytes/RFI of unstimulated lymphocytes) ×100.

### Single-cell Ca^2+^ Video Imaging

Fura-2/AM loaded T cells (1 µM, Invitrogen, Cergy-Pontoise, France) for 1 h at room temperature in HBSS (Invitrogen) were resuspended in HBSS 1%FCS and seeded on Lab-Tek glass chamber slides (Nunc, Naperville, IL) coated with poly-L-lysin (Sigma-Aldrich). Target cells were left to adhere on glass slides before addition of T cells. Measurements of intracellular Ca^2+^ responses were performed at 37°C with a DMI 6000 B microscope (Leica Microsystems, Nanterre, France). Cells were illuminated every 15 s with a 300 W xenon lamp by using 340/10 nm and 380/10 nm excitation filters. Emission at 510 nm was used for analysis of Ca^2+^ responses and captured with a Cool Snap HQ2 camera (Roper, Tucson, AZ) and analyzed with Metafluor 7.1 imaging software (Universal Imaging, Downington, PA).

### ^51^Cr Release Assay

Target cells were labeled with 100 µCi Na^51^CrO_4_ (Oris Industrie, Gif-sur-Yvette, France) for 1 h at 37°C, and incubated 4 h at 37°C, with effectors T cells at various E/T ratios. Then, 25 µl of supernatants were mixed with 100 µl of scintillation liquid (Optiphase Supermix, Wallak, United Kingdom) for measurement of radioactive content on a bêta plate counter (EG&G Wallac, Evry, France). Percentage of target cell lysis was calculated according to the following formula: [(experimental release – spontaneous release)/(maximum release – spontaneous release)]×100. Maximum and spontaneous releases were determined by, respectively, adding 0.1% Triton X-100 or medium to ^51^Cr-labeled target cells in the absence of T cells.

### Redirected Cytolytic Activity

1×10^3^ ^51^Cr-labeled murine mastocytoma FcγR P815 cells were incubated with T cells at various E/T ratio, in the presence of different concentrations of anti-CD3 Ab (clone OKT3). CD3 redirected lysis of P815 cells was modulated by the presence of indicated anti-NKR Abs (10 µg/ml). After 4 h, measurement of radioactive content and determination of percentage of specific lysis were performed.

## Results

### Frequency and Phenotypic Characteristics of HLA-E-reactive CD8 T Cells Isolated from Peripheral Blood of a Cytomegalovirus-seropositive Kidney-transplant Patient

**Figure 1:**
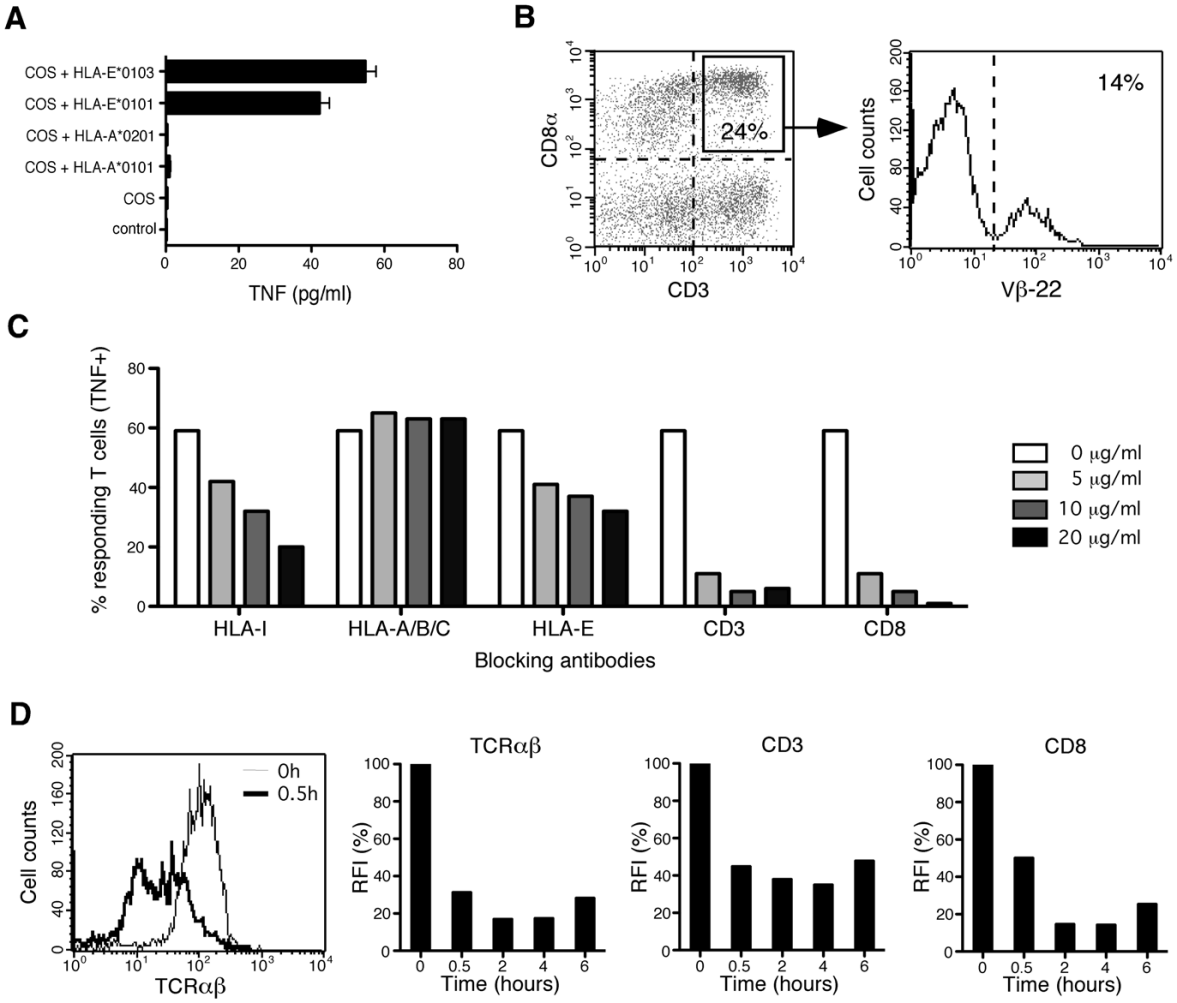
Characterization of HLA-E-restricted T cells in PBL of a kidney transplant patient undergoing CMV infection. A/Reactivity of freshly isloated PBL against COS-7 cells transfected, or not, with HLA-I encoding cDNA was assessed by a TNF release assay. Means and standard deviations of sixplicates are shown. B/Frequency of TCRVβ22^+^ HLA-E-restricted T cells in PBL. Fresh PBL were analyzed by three-color flow cytometry using antibodies specific for CD3, CD8a and Vβ22 TCR. Percentage of Vβ22 TCR expressing cells was examined on gated CD3^+^CD8^+^ T cells. C/TNF production in response to HLA-E transfected.221-E cells in the presence of blocking antibodies. MART.22 was stimulated with target cells in the presence or not of blocking antibodies directed against total HLA-I, HLA-A/-B/-C, HLA-E, CD3 and CD8 molecules at the indicated concentrations. After 6 h, T cells were fixed, permeabilized and stained for intracellular TNF-α. Results are expressed as percentage of TNF-producing T cells. D/Time course of TCRαβ, CD3 and CD8 surface expression on HLA-E-restricted CD8 T cells stimulated with.221-E cells. A representative FACS analysis of TCRαβ at early time course is shown (left panel). Results are expressed as percentages of RFI (as defined in Material and Methods).

Investigations of a cohort of renal transplant recipients [12] allowed us to identify an HLA-E-reactive CD8^+^ T cell population in PBL of a kidney transplant recipient with an active CMV infection. This HLA-E-restricted response was not observed on blood samples harvested before CMV infection (at one month post-transplantation) but appeared correlated with CMV infection 2 years post-transplantation, in associaton with a T cell response to pp65_495-503_/A*0201 HCMV epitope. As shown in Figure 1A, recipient fresh PBL activity, assessed by TNF-α production, was observed against COS-7 cells transfected with either HLA-E*0101 or HLA-E*0103 alleles whereas no response was observed with other HLA-I alleles tested. The HLA-E-reactive population was enriched and cloned. All the CD8 T cell clones derived (n = 9) were HLA-E-reactive and characterized by the homogeneous expression of the TCRVβ22 (data not shown). Notably, TCRVβ22^+^ cells represent a sizable fraction (3,4%) of freshly isolated recipient PBL, comprising 7% of CD3^+^ T cells and 14% of CD8^+^CD3^+^ T cells (Figure 1B). This monoclonal population, thereafter named MART.22, is characterized by CD8αβ^+^CD62L^-^CCR7^-^CD27^−^CD28^+/−^CD45RA^lo^CD45RO^hi^CD57^−^ surface phenotype (Figure S1), suggesting that MART.22 belongs to the effector-memory cell compartment [38]. Moreover, MART.22 expresses CD56 consistent with the phenotype of HLA-E-restricted NK-CTL previously reported by the group of L. Moretta [13].

### Requirement of Co-engagement of TCR and CD8 for HLA-E-reactive CD8 T Cells

To further characterize MART.22, we used the 721.221 B-EBV cell line (.221), lacking classical HLA class I molecules and HLA-G expression, and the 721.221-E cell line (.221-E), which has been stably transfected with the cDNA encoding HLA-E*0101 together with the leader sequence peptide from HLA-B*08, that is required for HLA-E cell surface expression and stabilization [18]. The transfected.221-E cell line, that consistently expresses high levels of HLA-E (Figure S2), induced strong activation of MART.22, as assessed by TNF production (59% of TNF-α producing T cells) (Figure 1C, white bar), whereas.221 cells were not recognized.

To assess the contribution of T cell receptor and HLA-E interaction to target cell recognition, we performed antibody blocking experiments and TCR down-regulation analysis. A dose-dependent reduction of TNF-α producing T cells was observed in the presence of anti-CD3 (until 5% vs 59%), anti-HLAI molecules (W6/32, until 20% vs 59%) or anti-HLA-E molecules (3D12, until 2% vs 59%) blocking antibodies (Figure 1C). By contrast blocking antibody specific for HLA-A/B/C molecules (G46-2.6) had no inhibitory effect on this process. TCR implication was also confirmed by the significant down-regulation of surface CD3/TCR complex after MART.22 stimulation with 221-E cells (Figure 1D). Furthermore, using the same approaches, we showed the high degree of CD8 dependency of MART.22 (Figures 1C–D). Together, these data confirm HLA-E restriction of MART.22 and unveil its strong CD8 dependency.

### Peptide Specificity of HLA-E-restricted CD8 T Cells

**Figure 2:**
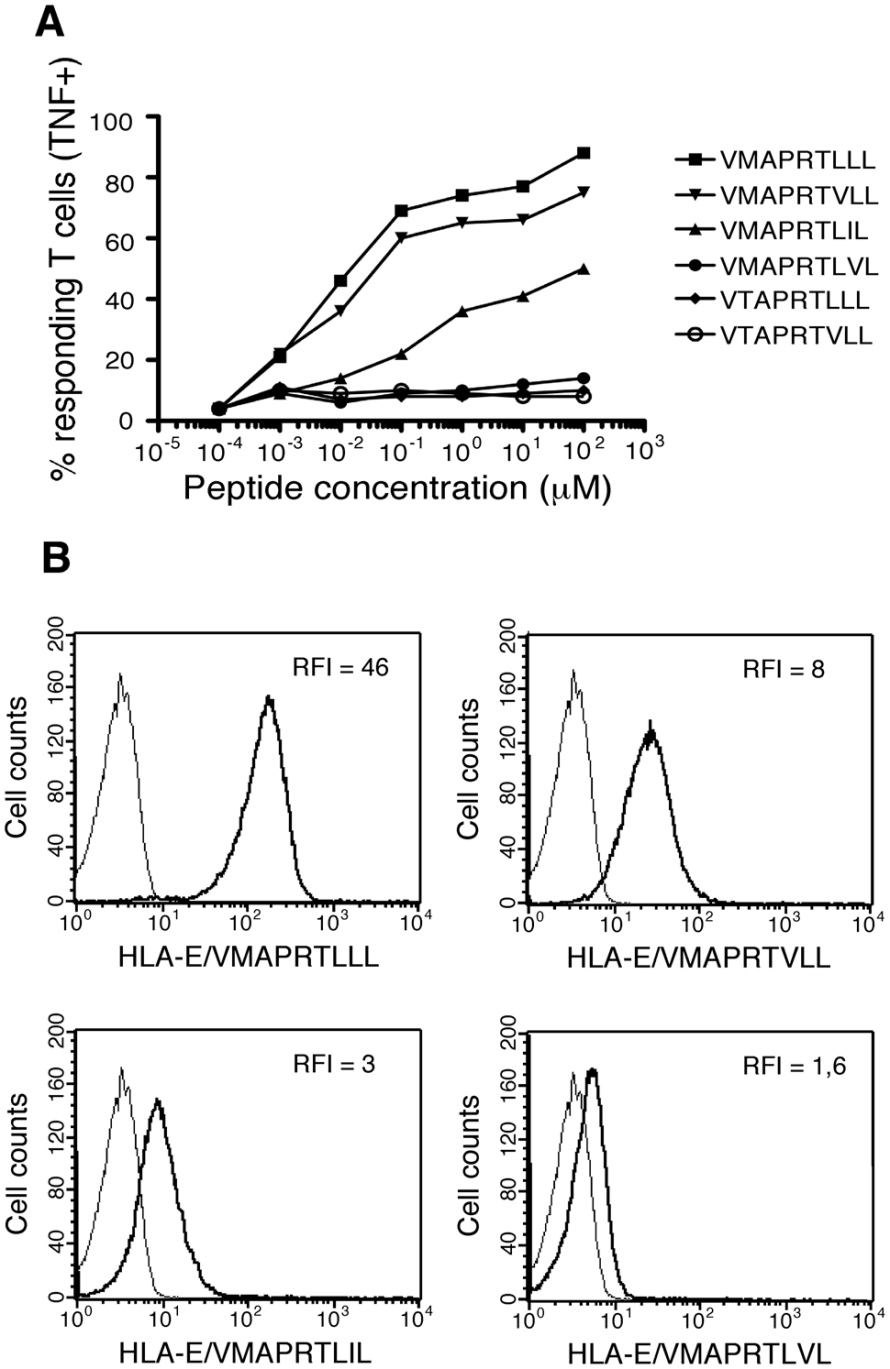
Characterization of CMV/HLA-I-derived peptides recognized by HLA-E-restricted CD8 T cells. A/TNF production in response to stimulation with.221 cells pulsed with synthetic peptides.221 cells were incubated for 1 h with range concentrations of the indicated peptides before addition of MART.22 T cells. After 6 h, T cells were fixed, permeabilized and stained for intracellular TNF-α. Results are expressed as percentage of TNF-producing T cells. B/Peptide-MHC tetramer staining of HLA-E-restricted CD8 T cells. MART.22 T cells were incubated for 1 h with biotyniled HLA-E monomers refolded with the indicated peptides and tetramerized with PE-coupled streptavidin. Peptide-HLA-E tetramers staining was assessed by flow cytometry and RFI are indicated.

Next, to investigate MART.22 peptide specificity, we test its ability to recognize.221 cells exogenously loaded with six HLA-E-restricted synthetic peptides (Table 1). This peptide set included the three previously described peptides derived from the UL40 protein of different human CMV strains [39], [40] and the peptides derived from the majority of HLA-I leader sequences, including autologous HLA-I from the transplant recipient. We found that MART.22 recognized.221 cells pulsed with 3 out of 6 peptides tested (Figure 2A). The VMAPRTLLL peptide was recognized with the highest avidity (EC50 at 1×10^−2^ µM). This peptide is derived from both the UL40 of the clinically isolate CMV 3C strain [39] and the leader sequence of various allogeneic HLA-A and HLA-C molecules. MART.22 also recognized with high avidity the VMAPRTVLL peptide (EC50 at 2×10^−2^ µM), which is derived from the leader sequence of various allogeneic HLA-B, including the HLA-B*08, molecules, thus providing explanation for the recognition of.221-E cells expressing HLA-B*08 leader sequence. MART.22 also recognized, albeit to a lesser extent (EC50 at 4×10^−2^ µM), the VMAPRTLIL peptide that derived from the UL40 of the laboratory CMV AD169 strain [39], [40]. This latter result was unexpected as this peptide also derives from the leader sequence of various HLA-C molecules, including the two autologous HLA-C alleles of the patient (ie HLA-Cw*1402 and -Cw*0501). The three other tested peptides (VTAPRTLLL, VTAPRTVLL and VMAPRTLVL) were not recognized at all, pinpointing to the importance of a methionine and of a leucine or an isoleucine at position 2 and 8 respectively to allow peptide recognition. To further substantiate our data on MART.22 peptide specificity, we used HLA-E*0101 tetramers refolded either with VMAPRTLLL, VMAPRTVLL, VMAPRTLIL or VMAPRTLVL peptides. As expected, Figure 2B shows the ability of MART.22 to bind HLA-E/VMAPRTLLL tetramers and to a lesser extent HLA-E/VMAPRTVLL and HLA-E/VMAPRTLIL tetramers whereas no significant binding was observed with tetramers refolded with the unrecognized VMAPRTLVL peptide.

**Table 1:** Leader sequence peptides derived from HCMV-UL40/HLA-I molecules and recognition by HLA-E-restricted T cell clone.

Leader sequence peptide_3–11_	HLA class I allotypes	MART.22 reactivity^a^
VMAPRTLVLb,c	HLA-A*02,-A*23, -A*24, -A*25, -A*26, -A*3402, -A*43, -A*66 and -A*69	**−**
VMAPRTLLL^b^	HLA-A*01,-A*03, -A*11, -A*29, -A*30, -A*31, -A*32, -A*33, -A*36 -A*74, -Cw*2 and -Cw*15	**+++**
VMAPRTLILb,c	HLA-Cw*01, -Cw*03, -Cw*0401, -Cw*05, -Cw*06, -Cw*0801-03, -Cw*12, -Cw*14,-Cw*16 and -Cw*1702	**+**
VMAPRTVLL	HLA-B*07, -B*08, -B*14, -B*38, -B*39, -B*42, -B*67, -B*73 and -B*81	**++**
VTAPRTLLL	HLA-B*13, -B*18, -B*27, -B*3542, -B*37, -B*40, -B*44, -B*47, -B*54, -B*56, -B*58, -B*59,-B*82 and -B*83	**−**
VTAPRTVLL	HLA-B*15, -B*35, -B*40, -B*41, -B*4418, -B*45, -B*49, -B*50, -B*51, -B*52, -B*57 and -B*78	**−**

Autologous HLA class I alleles of the transplant recipient are indicated in bold.

a MART.22 HLA-E-restricted T cell clone activity in response to.221 cells pulsed with different peptides (see Figure 2).

b These peptides are identical to peptides contained in the UL40 ORF from various CMV strains.

c These pepides have previously been described for their ability to trigger HLA-E restricted CD8 T cell responses.

These results show the ability of MART.22 to recognize peptides derived from both HCMV-UL40 and various allogeneic HLA-Ia molecules, suggesting that these cells may participate in the immune response against CMV-infected or allogeneic cells. HLA class I alleles of the kidney donor (HLA-A2, -B18, -B44 and -Cw5) are identical with the ones of the recipient (with the exception of HLA-B18 which give rise to an unrecognized peptide) therefore excluding that this population has been activated by the transplantation in favor to an induction as a consequence of CMV infection.

### Functional Characteristics of HLA-E-restricted CD8 T Cells

**Figure 3:**
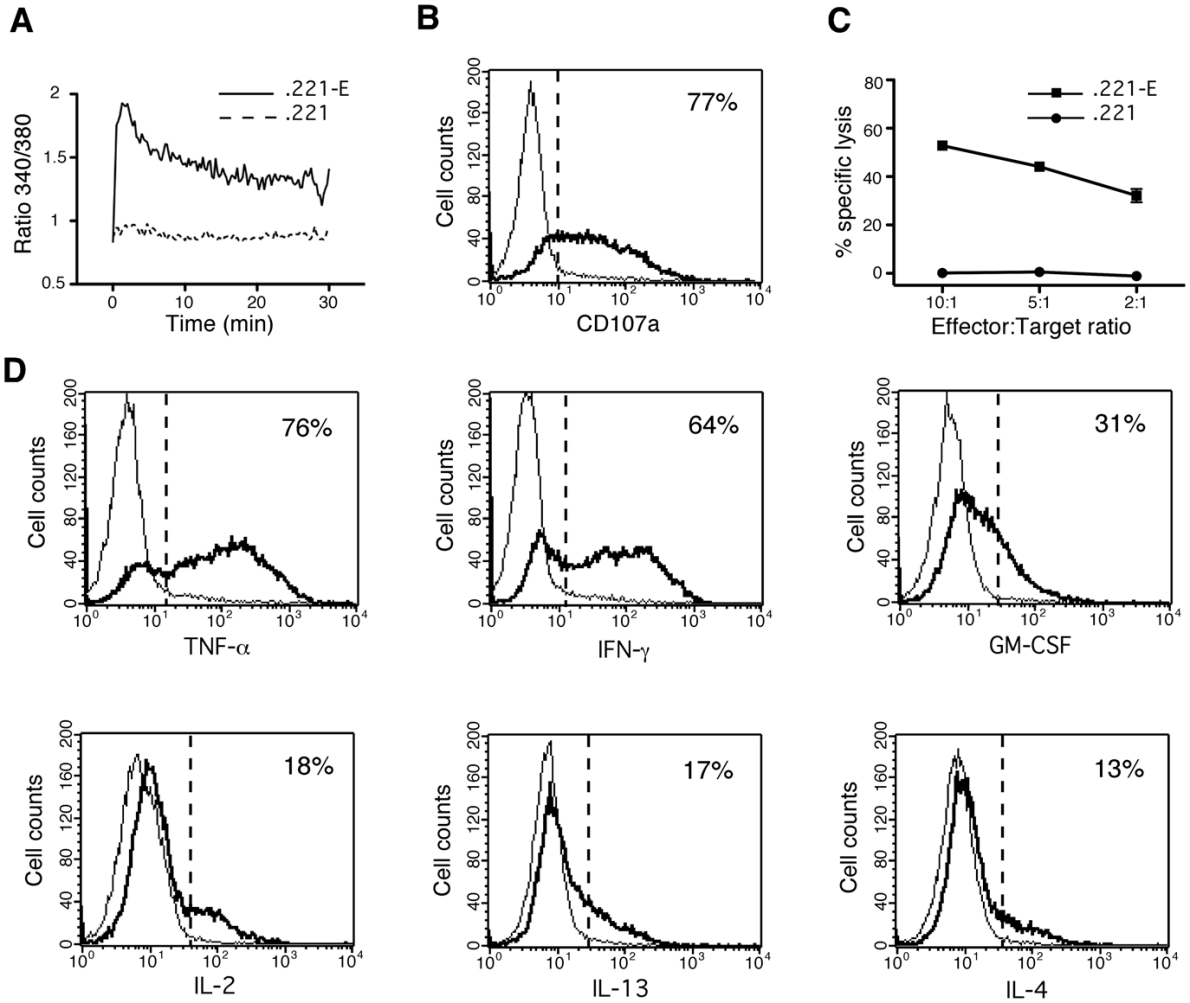
Functional characterization of HLA-E-restricted CD8 T cells. A/Induction of strong and rapid Ca^2+^ responses within activated HLA-E-restricted CD8 T cells. B-EBV 721.221 cells transfected (.221-E) or not (.221) with HLA-E and the leader sequence of HLA-B*08, were incubated with MART.22 T cells loaded with Fura-2 (1∶1 ratio). T cell intracellular Ca^2+^ levels were monitored by videomicroscopy for the indicated acquisition time. Graphs represent the kinetics of intracellular Ca2^+^ levels (340/380 nm ratio). Values correspond to the mean of emission measured among all T cells present in the field (approximatively 20 cells per experiment). Results are representative of two independent experiments. B/Degranulation of HLA-E-restricted CD8 T cells upon stimulation.221-E cells (thick line) or.221 cells (thin line) were incubated for 4 h with MART.22 T cells in the presence of anti-CD107a antibody. Results are expressed as pourcentages of surface CD107a positives T cells upon stimulation with.221-E cells. C/Cytotoxic activity of HLA-E restricted CD8 T cells. 10^3^ ^51^Cr-labeled.221-E cells (squares) or.221 cells (circles) were co-cultured for 4 h with MART.22 T cells at various E/T ratios. Cytotoxic activity was assessed through measure of Chromium release in the supernatants. Percentages of specific lysis are indicated. Means and standard deviations of triplicate wells are shown for one out of three comparable experiments. D/Cytokine production analysis of HLA-E restricted CD8 T cells. MART.22 T cells were fixed, permeabilized and stained for intracellular cytokines following 6 h of incubation with.221-E cells (thick line) or.221 cells (thin line). Data are expressed as mean % of intracellular cytokine secreting cells upon stimulation with.221-E cells.

Functional characterization of MART.22 was assessed using.221-E stimulating cells. As shown in Figure 3A, incubation with.221-E cells triggered a strong and rapid elevation in intracellular free calcium (Ca^2+^) concentration within MART.22 while no significant Ca^2+^ signal was detected when untransfected.221 cells were used. With regard to its potential ability to develop lytic response, incubation with.221-E cells induced MART.22 degranulation as demonstrated by the high CD107a surface mobilization (77% of CD107a positive T cells) and perforin/granzyme production (Figure 3B and data not shown). This leads to the lysis of.221-E cells as assessed with a standard ^51^Cr release assay (Figure 3C). As shown in Figure 3D, MART.22 was also found to produce high levels of TNF-α (78% of producing cells), IFN-γ (64%) and to a lower extent GM-CSF (31%), IL-2 (18%), IL-13 (17%) and IL-4 (13%). Conversely, no production of IL-5, IL-17F, IL-21, IL-22 or TGF-β was detected (data not shown). These data emphasize the strong granzyme-dependent cytolytic and TNF-α/IFN-γ secretion capacities of MART.22.

### Regulation of HLA-E-restricted CD8 T Cells Activity by NKR

**Figure 4:**
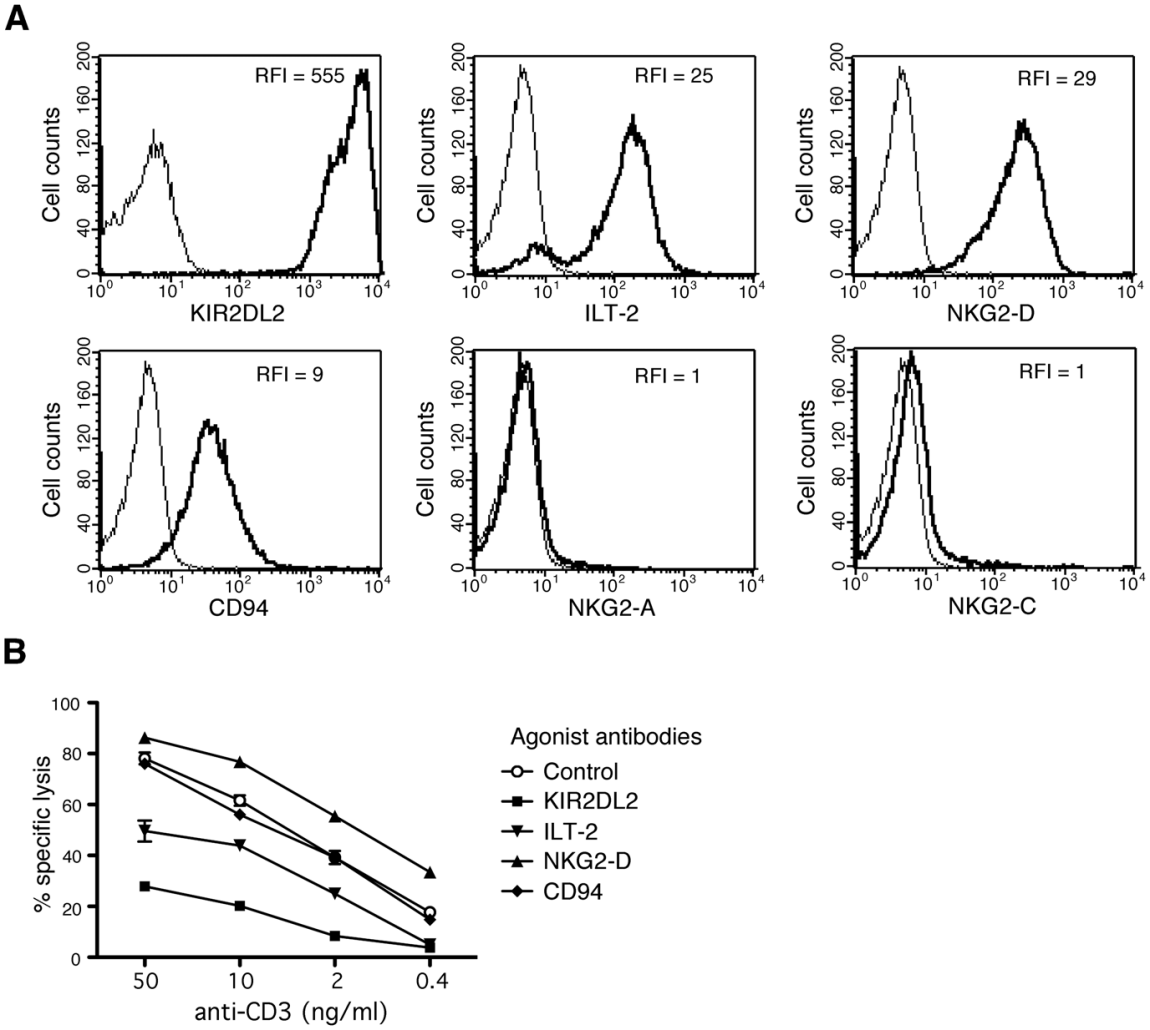
Expression of NK receptors by HLA-E-restricted CD8 T cells and functional characterization. A/Surface expression of NK receptors by HLA-E-restricted CD8 T cells. RFI of stained T cells (thick line) are indicated. B/Modulation of HLA-E restricted CD8 T cells reactivity through NKR engagement. ^51^Cr-labeled P815 cells were preincubated with the indicated concentration of anti-CD3 antibody in the presence or not of the indicated anti-NKR antibody for 1 h. Then, MART.22 T cells were added for 4 h. Redirected cytotoxic activity was assessed through measure of Chromium release in the supernatants. Percentages of specific lysis are indicated. Means and standard deviations of triplicate wells are shown for one representative experiments out of three performed.

As previous studies on HLA-E-restricted NK-CTL reported surface expression of HLA class I-specific inhibitory NK receptors (NKR), we investigated NKR expression on MART.22 (Figure 4A). MART.22 was strongly stained by the GL183 antibody, which recognizes KIR2DS2, KIR2DL2 and KIR2DL3. The combined use of KIR-specific mAbs [41] allowed us to identify the inhibitory KIR2DL2 as the KIR expressed by MART.22 (data not shown). Surface expression of ILT-2, NKG2-D and CD94 were also observed. Surprisingly, CD94 expression was not associated with NKG2-A or NKG2-C surface expression. In order to address the functionality of these receptors, we analyzed, in a redirected lysis assay, the ability of anti-NKR mAbs to modulate MART.22 TCR dependent lysis. As shown in Figure 4B, anti-CD3 mAb induced cytolytic activity was strongly inhibited by the addition of anti-KIR2DL2 mAb. Lysis was also inhibited, although to a lesser extent, by the addition of anti-ILT-2 mAb while it was slightly increased in presence of anti-NKG2-D mAb. However, addition of anti-CD94 mAb did not affect the lysis efficiency, clearly indicating the non-functionality of the CD94 receptor expressed by MART.22. Taken together, our data clearly indicate that the activity of HLA-E-restricted T cells can be modulated by competing positive or negative signals transduced by NKR, with especially efficient inhibition through KIR2DL2 ligation. Interestingly, autologous MART.22 HLA-C molecules (HLA-Cw*0501 and *1402) are ligands for the KIR2DL2 receptor [42]. Since these HLA-C molecules also provide a recognized HLA-E-bound peptide (Figure 2A and Table 1), this allowed us to hypothesize that inhibitory KIR2DL2 expression by MART.22 dampens its detrimental auto-reactivity against healthy (not CMV infected) autologous cells through ligation of autologous protective HLA-C molecules. Accordingly, when incubated in the presence of anti-KIR2DL2/DS2/DL3 or HLA-A/B/C blocking Abs, MART.22 developed fratricide response (Figure S3).

### HLA-E-restricted CD8 T Cells Reactivity Against Allogeneic Endothelial Cells

**Figure 5:**
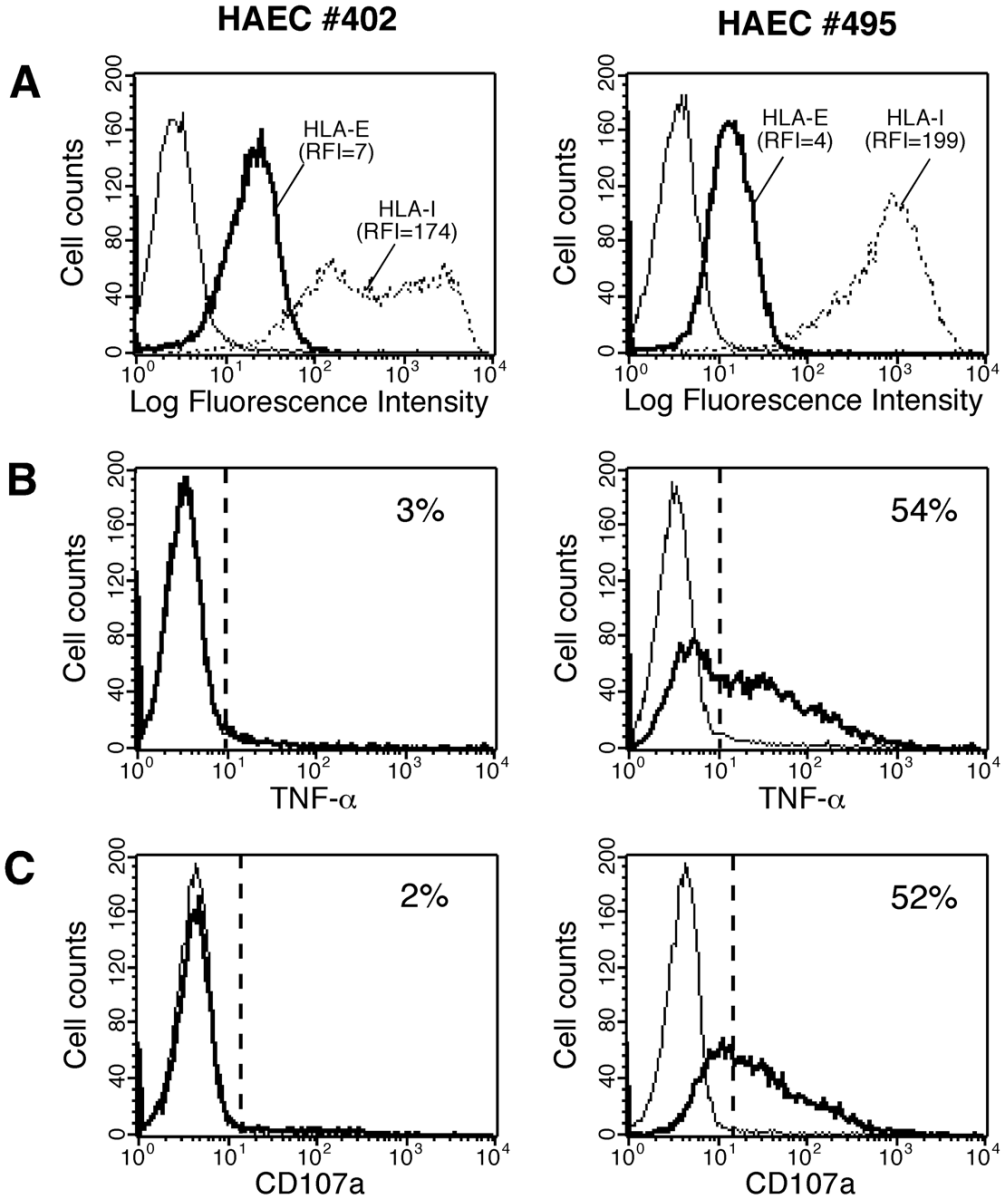
Reactivity of HLA-E-restricted CD8 T cells against allogeneic endothelial cells. A/Surface expression of HLA-E (thick lines) and total HLA-I (dotted lines) molecules by two representative endothelial cultures (HAEC). RFI are indicated. B/Cytokine production by HLA-E-restricted CD8 T cells upon stimulation with endothelials cultures. MART.22 T cells were fixed, permeabilized and stained for intracellular TNF-α following 6 h of incubation with HAECs (thick line) or not (thin line). Data are expressed as percentage of intracellular cytokine secreting T cells upon stimulation with HAECs. C/Degranulation of HLA-E-restricted CD8 T cells upon stimulation with endothelial cultures. MART.22 T cells were incubated for 4 h with HAECs (thick line) or not (thin line) in the presence of anti-CD107a antibody. Results are expressed as percentages of surface CD107a positive T cells upon stimulation with endothelial cells.

Since we demonstrated that peptides derived from both CMV-UL40 and allogeneic HLA-I molecules can be recognized by MART.22 in an HLA-E-restricted fashion, we asked whether MART.22 could also recognize and damage allogeneic endothelial cells and therefore represent a risk factor for allograft outcome. To this end, primary human arterial endothelial cell (HAEC) cultures, isolated from kidney transplant donors were tested *in vitro* for their capacity to activate MART.22. HLA-I typing of the seven endothelial cell cultures tested as well as their capacity to provide recognized peptides or to interact with KIR2DL2 are documented in Table 2. All EC cultures expressed HLA-I molecules carrying peptides potentially recognized in the HLA-E context. The CMV serologic status of EC donors is also indicated. While surface HLA-E staining levels were similar on all EC cultures tested (Figure 5A and data not shown), six out of seven EC cultures induced efficient cytokine responses of MART.22, as illustrated by TNF-α production (from 24% to 75% of T cells) (Figure 5B and Table 2). Moreover, MART.22 develops cytolytic responses against recognized endothelial cells, as assessed by CD107a surface expression (from 8% to 68% of T cells) (Figure 5C and Table 2). In accordance with recognition of both allelic forms of HLA-E by MART.22 (Figure 1A), endothelial cells are recognized independently of their HLA-E haplotype and with no correlation to CMV infection (mean value, 42% of TNF producing T cells for CMV negative versus 41% for CMV positive patients), suggesting the direct recognition of allogeneic HLA-I derived peptides in an HLA-E-restricted fashion. Thus, HLA-E-restricted T cells could represent a risk factor for allograft outcome through recognition of allogeneic graft endothelial cells.

**Table 2:** Characteristics of endothelial cells (HLA class I allotypes and HCMV serologic status of donors) and recognition by HLA-E-restricted T cell clone.

HAEC	HLA-Ia allotypes							HLA-E allotypes		HCMV sero-positivity	MART.22 reactivity^a^	
	HLA-A			HLA-B		HLA-Cw					TNF-α	CD107a
#112	*0201	*2402	*1801		*5101	*0202	*0701^b^	*0103	*0103	**+**	49%	32%
#116	*0201	*2902	*3501		*4402	*0401 c	*0501 b	*0101	*0101	**+**	24%	8%
#323	*0301	*2402	*4701		*5001	*0602	*0602	ND	ND	**−**	75%	68%
#331	*0301	*3201	*0702		*3701	*0602	*0702^b^	*0101	*0103	**−**	58%	41%
#337	*2402	*3101	*3501		*4001	*0401 c	*0304 b	*0103	*0103	**−**	26%	10%
#402	*2301	*2902	*4403		*5801	*0701^b^	*1601 b	*0101	*0103	**−**	3%	4%
#495	*0101	*0201	*4101		*4402	*0501 b	*1701	ND	ND	**+**	54%	52%

HLA-Ia alleles susceptible to provide peptides recognized by HLA-E-restricted T cell clone are indicated in bold.

a HLA-E-restricted T cell clone activity in response to endothelial cells (see Figure 5).

b HLA-C allotypes carrying the C1 epitope that are susceptible to bind to KIR2DL2 receptor.

c HLA-Cw0401 allotype that has been shown to interact with KIR2DL2 receptor.

### Tight Regulation of HLA-E-restricted CD8 T Cells Alloreactivity by KIR2DL2

**Figure 6:**
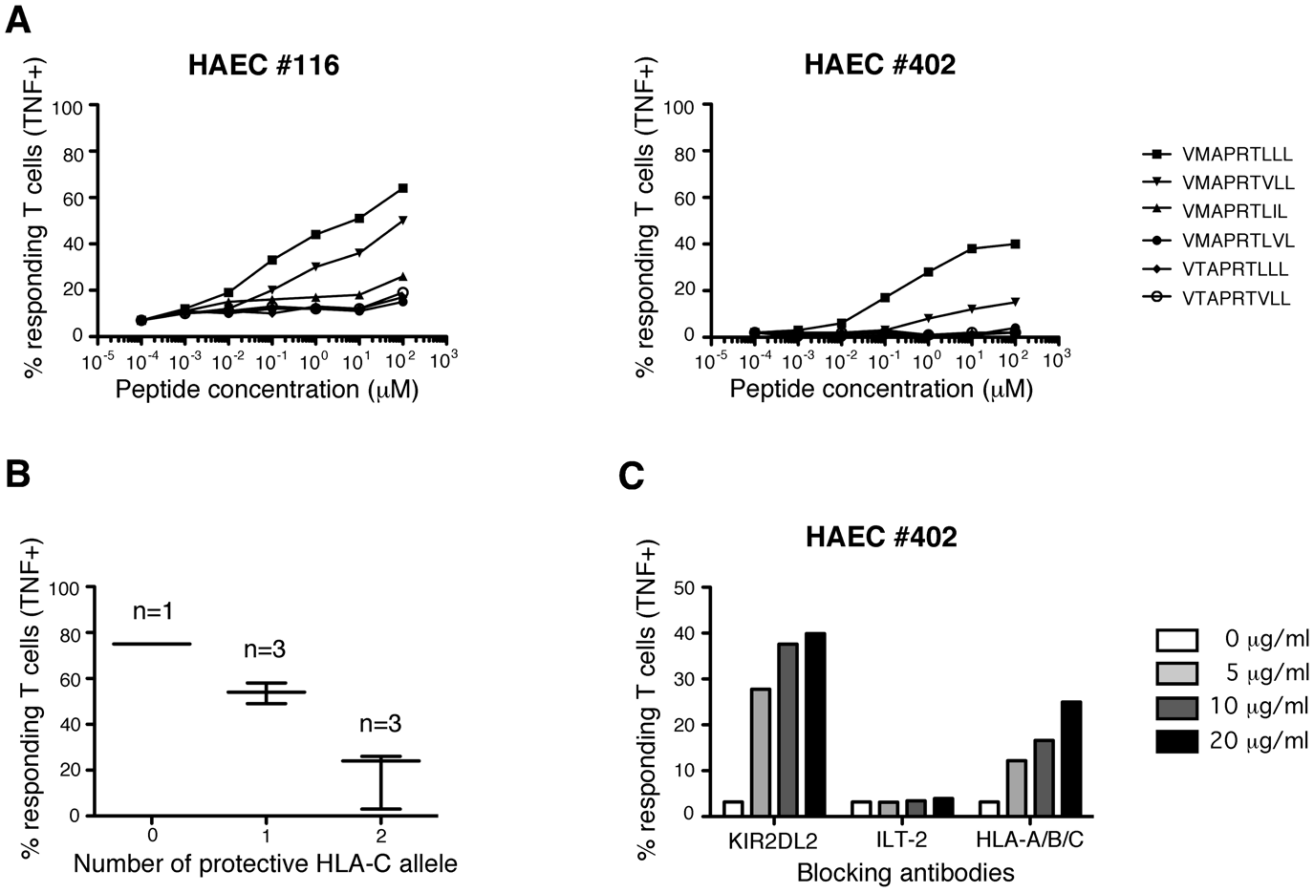
Regulation of HLA-E-restricted CD8 T cells reactivity against allogeneic endothelial cells by NK receptor. A/Reactivity of HLA-E-restricted T cells against poorly recognized (HAEC#116) or unrecognized (HAEC#402) endothelial cultures pulsed with synthetic peptides. HAECs were incubated for 1 h with range concentrations of the indicated peptides before MART.22 T cells were added. After 6 h, T cells were fixed, permeabilized and stained for intracellular TNF-α. Results are expressed as percentage of TNF-producing T cells. B/Impact of KIR2DL2-ligands expression by HAECs on HLA-E-restricted T cells alloreactivity. Percentages of TNF-producing MART.22 T cells are shown for HAECs with none, one or two protective HLA-C alleles. C/Reactivity of HLA-E-restricted T cells against unrecognized endothelial cultures (HAEC#402) in the presence of blocking antibodies. HAECs were incubated with MART.22 T cells in the presence or not of indicated concentrations of blocking antobodies. After 6 h, T cells were fixed, permeabilized and stained for intracellular TNF-α. Results are expressed as percentage of TNF-producing T cells.

As mentioned above, in an unexpected way, one EC culture (HAEC#402), with no apparent defect in surface HLA-E expression levels, was not recognized by MART.22 (Figure 5A). To ascertain this was not the consequence of the specific lack of expression of HLA-I molecules encoding recognized peptides, we investigated whether incubation with the two best-recognized synthetic peptides could render these endothelial cells more susceptible to recognition by MART.22. As shown on Figure 6A, pulsing of the otherwise resistant HAEC#402 with VMAPRTLLL and VMAPRTVLL induced TNF-α production by MART.22 but only with saturating amounts of peptides (respectively 40% and 18% of TNF secreting T cells when HAEC#402 were loaded with 10^2^ µM of peptides). Similar results were obtained with another poorly recognized EC culture (HAEC#116), suggesting another mechanism conferring resistance to recognition. As we showed that MART.22 reactivity is strongly regulated by the inhibitory KIR2DL2, we investigated whether HAEC suboptimal recognition was indeed the consequence of the expression of protective HLA-C molecules (ie KIR2DL2 ligands) [42], [43], [44]. Interestingly, HLA-C haplotype crucially influence the MART.22 alloreactivity: endothelial cells possessing two appropriate HLA-C alleles (HAEC#116, #337 and #402) are less recognized (mean value, 18% of TNF producing T cells) than those bearing only one (HAEC#112, #331 and #495, 54% of TNF producing T cells) or no (HAEC#323, 75%) (Figure 6B). This was confirmed by assessing the effect of blocking antibodies on endothelial cells recognition by MART.22. As shown on Figure 6C, addition of KIR2DL2-blocking Abs and, to a lesser extent, of anti-HLA-A/B/C Abs efficiently restore the HAEC#402 recognition by MART.22 in a dose dependent manner (up to 40% and 25% respectively), whereas addition of blocking Ab to ILT-2 had no significant effect. These results underline the tight regulation of HLA-E-restricted allo-reactivity by KIR2DL2 receptors through their recognition of HLA-C molecules expressed on target cells.

**Figure 7:**
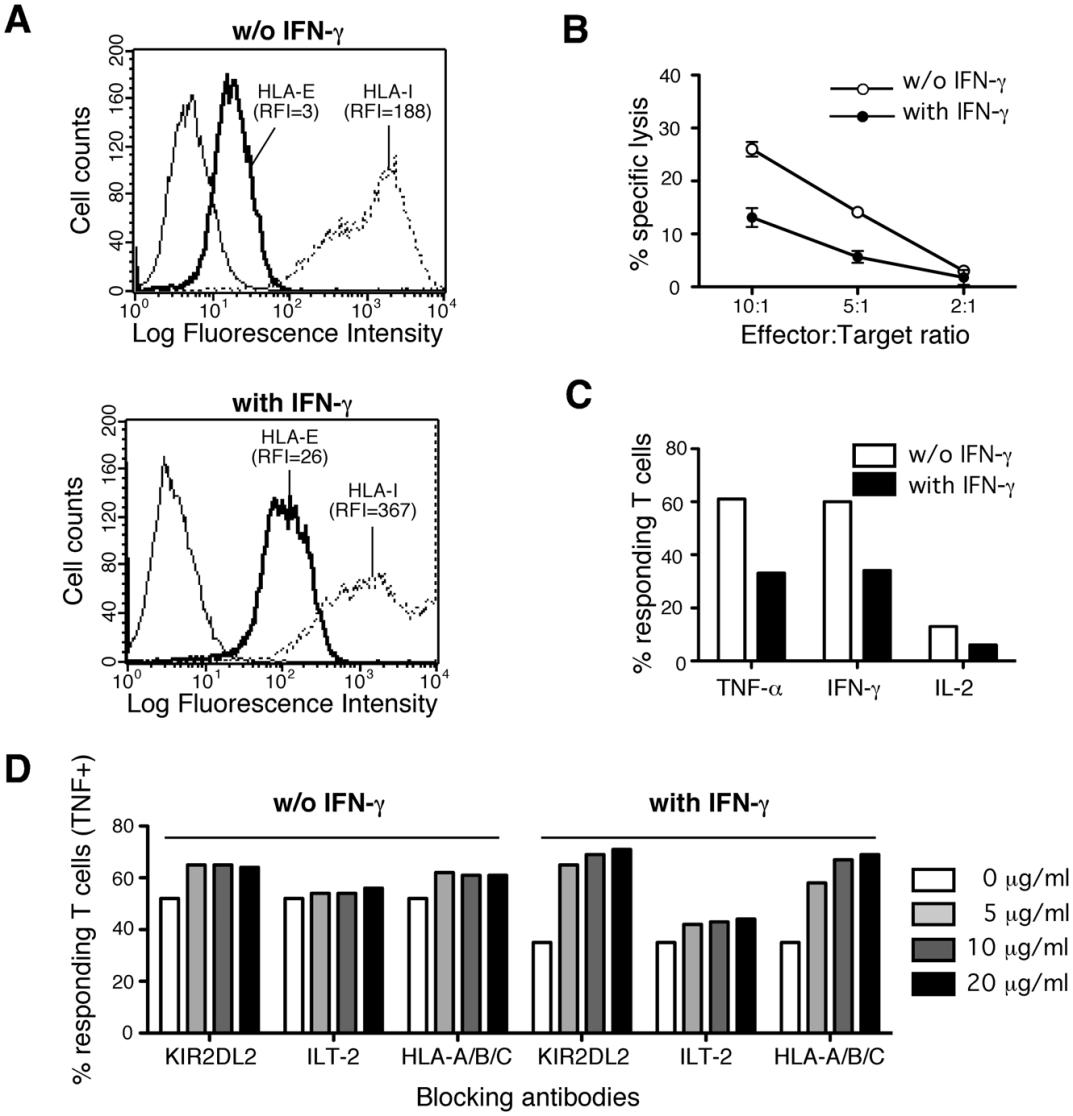
Impact of IFN-γ treatment on allogeneic endothelial cells (HAEC#495) **recognition by HLA-E-restricted CD8 T cells.** A/Impact of IFN-γ treatment on surface expression of HLA-E (thick lines) and total HLA-I (dotted lines) molecules by endothelial cultures. RFI are indicated. B/HLA-E-restricted CD8 T cells cytotoxicity toward endothelial cultures treated or not with IFN-γ. 10^3^ ^51^Cr-labeled HAECs pretreated (closed circles) or not (open circle) with IFN-γ were co-cultured for 4 h with T cells at various E:T ratio. Cytotoxic activity was assessed trought measure of Chromium release in the supernatants. Percentages of specific lysis are indicated. Means and standard deviations of triplicate wells are shown for one representative experiments out of three performed. C/HLA-E-restricted CD8 T cells cytokine production upon stimulation with endothelials cultures treated or not with IFN-γ. MART.22 T cells were fixed, permeabilized and stained for intracellular cytokines following 6 h of incubation with HAECs pretreated (black bars) or not (white bars) with INF-γ. Data are expressed as percentages of intracellular cytokine secreting T cells upon stimulation. D/HLA-E-restricted CD8 T cells cytokine production upon stimulation with endothelials cultures treated or not by IFN-γ, in the presence of blocking antibodies. MART.22 T cells were fixed, permeabilized and stained for intracellular cytokines following incubation for 6 h with HAECs pretreated or not with IFN-γ in the presence or not of various amount of blocking antibodies directed against KIR2DL2, ILT-2 and HLA-Ia molecules. Data are expressed as percentages of intracellular TNF secreting T cells upon stimulation.

### Effect of IFN-γ Treatment on Endothelial Cells Recognition by HLA-E-restricted CD8 T Cells

Chronic CMV infections result in recruitment of inflammatory cells and mediators such as chemokines and cytokines including IFN-γ [4]. So, we analyzed the impact of IFN-γ treatment of EC cultures on their recognition by MART.22. As we previously reported [30], IFN-γ treatment enhances both HLA-E and total HLA-I surface expression on endothelial cells (Figure 7A and Figure S4A). However, IFN-γ treatment of endothelial cells resulted in decreased MART.22 mediated lysis and cytokine production (Figures 7B–C and Figures S4B–C). The percentage of TNF-α producing T cells upon stimulation with the HAEC#495 fell from 61% to 33% after IFN-γ treatment. Experiments performed with a less recognized EC culture show that MART.22 reactivity against IFN-γ treated HAEC#116 was completely abolished. To investigate whether the inhibitory effect of IFN-γ treatment was the consequence of an increased expression of inhibitory NKR ligands by endothelial cells, we performed antibody blocking experiments. First, anti-KIR2DL2 and anti-ILT-2 antibodies had little or no effect on recognition of the untreated HAEC#495 culture. In contrast, these antibodies, especially the anti-KIR2DL2 mAb, improved in a dose dependent manner the recognition of IFN-γ treated endothelial cells (71% vs 36% of TNF-α producing T cells for the maximal dose of anti-KIR2DL2 Ab) (Figure 7D). In the same way, mAb directed against classical HLA-I molecules, which are ligands of both KIR2DL2 and ILT-2, greatly enhanced recognition of IFN-γ treated endothelial cells recognition (69% vs 36% of TNF-α producing T cells for the maximal dose of Ab). Taken together, these data underline the crucial role of inhibitory NKR ligands which expression on EC is a determining factor for HLA-E-restricted T cells reactivity.

## Discussion

In conclusion, this study demonstrates for the first time the ability of CMV-associated HLA-E-restricted T cells from transplant recipient to recognize and lyse allogeneic endothelial cells thereby emphasizing their potential detrimental alloreactivity upon solid organ transplantation.

A function for HLA-E as a restricting element for the TCR of αβ T cells has been clearly established [21] and therefore can play a role in the adaptive immune response in addition to its well-known regulation of innate immunity [45], [46]. The HLA-E-restricted CD8 αβ T cell population described in this study appears in association with a T cell response to classical HLA I-restricted HCMV epitope (pp65/A*02) in the blood of a kidney transplant recipient with an active CMV infection. Thus, HLA-E-restricted T cells may be induced *in vivo* in recipient patients as a consequence of CMV infection or reactivation, suggesting their possible role in the immune adaptative response to CMV. Various CMV proteins inhibit MHC class Ia surface expression impeding the control mediated by conventional (i.e. MHC class Ia-restricted) CD8 T cells [47], [48]. Therefore, the capacity of CMV, through the expression of UL40, to supply HLA-E-binding peptides allowing increase of HLA-E surface expression in infected cells [40], strengthen that HLA-E-restricted T cells may have a particular relevance in the immune defense against CMV.

In accordance with previous studies showing that CMV-associated HLA-E-restricted T cells represent a pauciclonal population comprising a sizable fraction of CD8 αβ T cells in CMV-seropositive patients [15], [49], the population described in this study expresses homogeneously a given TCR owing to its monoclonal origin and constitutes a significant component of peripheral blood mononuclear cells (14% of CD8^+^CD3^+^ T cells). Moreover, we showed that this population has phenotypic characteristics of effector-memory lymphocytes and displays strong granzyme-dependent cytolytic and TNF-α/IFN-γ secretion capacities, suggesting that they could play a relevant role in the control of CMV infection.

As three different HLA-E-binding HCMV-UL40-derived peptides have been previously described, we investigated the specificity of our HLA-E-restricted T cells. Previous studies from the group of L. Moretta have characterized HLA-E-restricted T cells reacting against peptides (i.e. VMAPRTLIL and VMAPRTLVL) derived from the UL40 of 2 HCMV laboratory strains (Toledo and AD169 strains) [15]. The HLA-E-restricted T cell population described here reacts against the additional UL40 derived-peptide, VMAPRTLLL, that has been shown to derive from the clinical isolate HCMV 3C strain.

Because recognized peptides also derived from the leader sequences of numerous allogeneic HLAI alleles, CMV-associated HLA-E-restricted T cells have potential to mediate allograft rejection through direct recognition of allogeneic HLA-I derived-peptides presented by HLA-E on graft cells. In a previous study, we showed that HLA-E protein expression in normal human organs is mainly restricted to endothelial cells and leucocytes [30]. Hence, owing to the crucial role of endothelial cells in allo-antigen presentation to T cells [8] and to the HCMV tropism for endothelial cells [5], [7], we investigated whether HLA-E-restricted T cells could recognize primary endothelial cells cultures, isolated from kidney allografts. We clearly demonstrate that CMV-associated HLA-E-restricted CD8 T cells can efficiently recognized and killed allogeneic endothelial cells *in vitro*, independently of their HLA-E allotype. Therefore, because immunosuppressed transplant patients are particularly prone to CMV infection, we can speculate that in the context of both CMV reactivation or primary infections, while these T cells have potential to contribute to infection control, they may also directly recognize allogeneic graft endothelial cells and thereby contribute to allograft rejection.

As suggested by previous studies, we clearly demonstrated that CMV-associated HLA-E-restricted T cell allo-reactivity is tightly regulated by NK receptors [50]. We first showed surface expression of KIR2DL2, ILT-2, NKG2D and CD94 receptors by MART.22. Surprisingly, CD94 surface expression was not associated with that of NKG2-A or NKG2-C molecules and did not allow interaction with HLA-E tetramer refolded with HLA-A2 peptide, suggesting the expression of CD94 homodimers as previously described [51]. Finally, we demonstrated the non-functionality of this receptor. All the other expressed NK receptors were found to be functional, with a predominant role in preventing target cell recognition for the highly expressed inhibitory KIR2DL2 through ligation of appropriated (protective) HLA-C molecules [42]. The expression of KIR2DL2 appears to constitute a safety mechanism avoiding harmful autoreactivity through the ligation of protective autologous HLA-C molecules. As a consequence, the ability of HLA-E-restricted T cells to mediate alloreactivity against endothelial cells was crucially impacted by the expression of protective HLA-C alleles. Thus, allogeneic endothelial cells that express protective HLA-C molecules, or that were pre-treated with INF-γ, were less recognized by HLA-E-restricted T cells, unless specific blocking antibodies (i.e. anti-KIR2DL2 or anti-HLA-A/B/C) were added to the cultures. This underlines the crucial impact of HLA-C haplotype of target cells on their ability to trigger, or not, an allogeneic HLA-E-restricted T cell response. Therefore, HLA-C haplotypes that are still underestimated in transplantation setting should be reconsidered and taken into account [52], [53].

In conclusion, we demonstrated, for the fist time, that CMV infection in transplant patient correlated with an allo-reactive HLA-E-restricted T cell response that have potential to mediate detrimental vascularized allograft rejection via endothelial cells lysis. Therefore, CMV-associated HLA-E restricted T cells could account for the well-established association between CMV-infection and accelerated allograft rejection. As HLA-E is also expressed in leucocytes, the involvement of HLA-E-restricted T cells in the immunological response following allogeneic hematopoietic stem cell transplantation should also be addressed, as it has been suggested by studies using transgenic mice [32]. Moreover, we provided strong evidence that HLA-C/NKR mismatch is a key player in HLA-E-restricted T cells alloreactivity. Thus, graft organ HLA-C haplotypes may impact on CMV-associated HLA-E-restricted T cells capacity to mediate allograft rejection. Hence, a deeper evaluation of the frequency and the role of CMV-associated HLA-E-restricted T cells in transplantation and of the impact of HLA-C haplotype on their alloreactivity, may determine whether this indeed represents an additional risk factor following solid organ transplantation.

## Supporting Information

Figure S1 **Phenotypic characterization of HLA-E-restricted T cells.** The surface expression of various markers was assessed by flow cytometry and the RFI plotted graphically.
(TIF)

Figure S2 **Surface expression of HLA-I molecules by B-EBV cell lines transfected (.221-E) or not (.221) with HLA-E.** Surface expression of total HLA-I (A), HLA-Ia (B) and HLA-E (C) molecules. RFI are indicated.
(TIF)

Figure S3 **Autoreactivity of HLA-E-restricted T cells in the presence of blocking antibodies.** MART.22 T cells were incubated in the presence (thick lines) or in the absence (thin lines) of blocking antobodies (10 µg/ml) directed agains KIR2DL2 (A), ILT-2 (B) and HLA-Ia (C). After 6 h, T cells were fixed, permeabilized and stained for intracellular TNF-α. Results are expressed as percentage of TNF-producing T cells when incubated with blocking antibodies.
(TIF)

Figure S4 **Impact of IFN-γ treatment on allogeneic endothelial cells (HAEC#116)** **recognition by HLA-E-restricted CD8 T cells.** A/Impact of IFN-γ treatment on surface expression of HLA-E (thick lines) and total HLA-I (dotted lines) molecules by endothelial cultures. RFI are indicated. B/HLA-E-restricted CD8 T cells cytotoxicity toward endothelial cultures treated or not with IFN-γ. 10^3^ ^51^Cr-labeled HAECs pretreated (closed circles) or not (open circle) with IFN-γ were co-cultured for 4 h with T cells at various E:T ratio. Cytotoxic activity was assessed trought measure of Chromium release in the supernatants. Percentages of specific lysis are indicated. Means and standard deviations of triplicate wells are shown for one representative experiments out of three performed. C/HLA-E-restricted CD8 T cells cytokine production upon stimulation with endothelials cultures treated or not with IFN-γ. MART.22 T cells were fixed, permeabilized and stained for intracellular cytokines following 6 h of incubation with HAECs pretreated (black bars) or not (white bars) with INF-γ. Data are expressed as percentages of intracellular cytokine secreting T cells upon stimulation.
(TIF)
