# Supplementary figures and images for: HLA-E-Restricted Cross-Recognition of Allogeneic Endothelial Cells by CMV-Associated CD8 T Cells: A Potential Risk Factor following Transplantation
Source: PLoS One. 2012 Nov 30;7(11):e50951. doi: 10.1371/journal.pone.0050951 (PMC3511380; doi:10.1371/journal.pone.0050951)

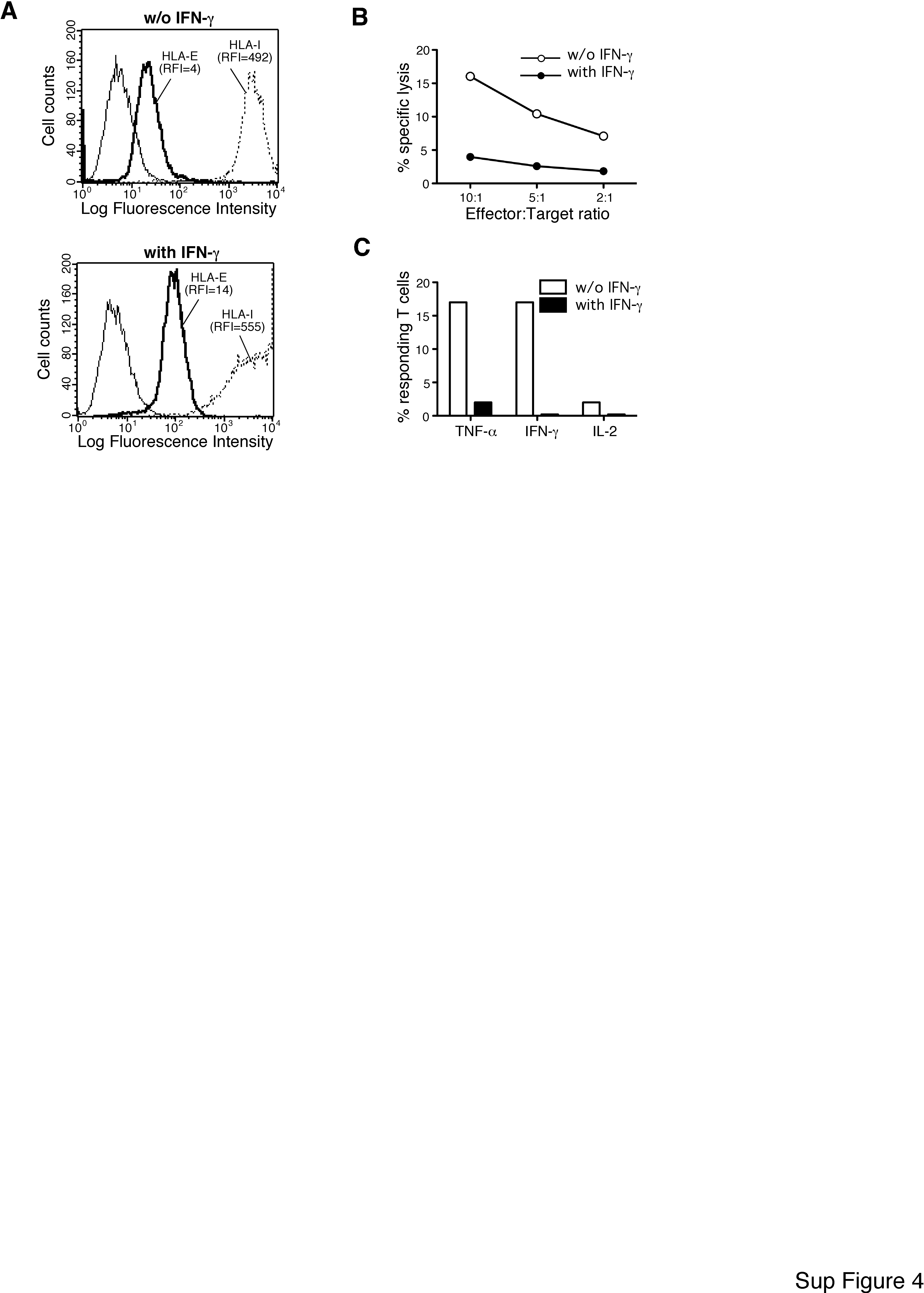

Supplement: Figure S4 — Impact of IFN-γ treatment on allogeneic endothelial cells (HAEC#116) recognition by HLA-E-restricted CD8 T cells. A/Impact of IFN-γ treatment on surface expression of HLA-E (thick lines) and total HLA-I (dotted lines) molecules by endothelial cultures. RFI are indicated. B/HLA-E-restricted CD8 T cells cytotoxicity toward endothelial cultures treated or not with IFN-γ. 103 51Cr-labeled HAECs pretreated (closed circles) or not (open circle) with IFN-γ were co-cultured for 4 h with T cells at various E:T ratio. Cytotoxic activity was assessed trought measure of Chromium release in the supernatants. Percentages of specific lysis are indicated. Means and standard deviations of triplicate wells are shown for one representative experiments out of three performed. C/HLA-E-restricted CD8 T cells cytokine production upon stimulation with endothelials cultures treated or not with IFN-γ. MART.22 T cells were fixed, permeabilized and stained for intracellular cytokines following 6 h of incubation with HAECs pretreated (black bars) or not (white bars) with INF-γ. Data are expressed as percentages of intracellular cytokine secreting T cells upon stimulation. (TIF) [file pone.0050951.s004.tif]
